# Supplementary material for: From sequence to enzyme mechanism using multi-label machine learning
Source: BMC Bioinformatics. 2014 May 19;15:150. doi: 10.1186/1471-2105-15-150 (PMC4229970; doi:10.1186/1471-2105-15-150)
Supplement: Additional file 2 — Java code of ml2db. Additional file ml2db_code.tar.gz contains the Java source code to run the multi-label machine learning experiments and save the results to database. The code’s Javadoc is included. [file 1471-2105-15-150-S2.zip › additional file 2/ml2db/ml2db/doc/index-files/index-18.html]

R-Index


JavaScript is disabled on your browser.


- Overview
- Package
- Class
- Use
- Tree
- Deprecated
- Index
- Help

- Prev Letter
- Next Letter

- Frames
- No Frames

- All Classes

A B C D E F G H I J K L M N O P Q R S T U V W X 


## R

RandomAttributePicker - Class in main.ml2db.al.utility.pickers
:   Picks an attribute uniformly randomly and then one of the unlabelled
    instances having that attribute.

RandomAttributePicker() - Constructor for class main.ml2db.al.utility.pickers.RandomAttributePicker


RandomAttributePickerTest - Class in test.ml2db.al.utility.pickers
:   Class

RandomAttributePickerTest() - Constructor for class test.ml2db.al.utility.pickers.RandomAttributePickerTest


RandomAttributeSetPicker - Class in main.ml2db.al.utility.pickers
:   Class

RandomAttributeSetPicker() - Constructor for class main.ml2db.al.utility.pickers.RandomAttributeSetPicker


RandomAttributeSetPickerTest - Class in test.ml2db.al.utility.pickers
:   Class

RandomAttributeSetPickerTest() - Constructor for class test.ml2db.al.utility.pickers.RandomAttributeSetPickerTest


RandomInstancePicker - Class in main.ml2db.al.utility.pickers
:   Class

RandomInstancePicker() - Constructor for class main.ml2db.al.utility.pickers.RandomInstancePicker


RandomLabelsPicker - Class in main.ml2db.al.utility.pickers
:   Class

RandomLabelsPicker() - Constructor for class main.ml2db.al.utility.pickers.RandomLabelsPicker


RandomNeigbourhoodPicker - Class in main.ml2db.al.utility.pickers
:   Class

RandomNeigbourhoodPicker() - Constructor for class main.ml2db.al.utility.pickers.RandomNeigbourhoodPicker


RandomPicker - Class in main.ml2db.al.utility.pickers
:   Class

RandomPicker() - Constructor for class main.ml2db.al.utility.pickers.RandomPicker


readSource(String) - Method in class main.ml2db.graphviz.GraphViz
:   Read a DOT graph from a text file.

rebuild(IdMultiLabelInstances) - Method in class main.ml2db.al.utility.neighbours.LinearMultiLabelNNSearch


recordArff() - Method in class main.ml2db.weka.ImportArff


refresh(IdMultiLabelInstances, IdMultiLabelInstances, IdInstance) - Method in class main.ml2db.al.utility.confidence.ConfidenceMetric


refresh(IdMultiLabelInstances, IdMultiLabelInstances, IdInstance) - Method in class main.ml2db.al.utility.globaldistance.GlobalDistanceMetric


refresh(IdMultiLabelInstances, IdMultiLabelInstances, IdInstance) - Method in class main.ml2db.al.utility.hybrid.GlobalDistanceWithConfidenceMetric


refresh(IdMultiLabelInstances, IdMultiLabelInstances, IdInstance) - Method in class main.ml2db.al.utility.neighbours.NeighbourhoodNetwork


refresh(IdMultiLabelInstances, IdMultiLabelInstances, IdInstance) - Method in class main.ml2db.al.utility.pickers.byfrequency.NearestNeighboursByFreqUtility


refresh(IdMultiLabelInstances, IdMultiLabelInstances, IdInstance) - Method in class main.ml2db.al.utility.pickers.RandomPicker


refresh(IdMultiLabelInstances, IdMultiLabelInstances, IdInstance) - Method in class main.ml2db.al.utility.UtilityMetric
:   Refresh the set of labelled and unlabelled instances

refreshConnection() - Method in class main.ml2db.DbManagers


resetEvaluation() - Method in class main.ml2db.al.learning.AlOutputManager


ResultCheckerTest - Class in test.ml2db.mulan.learn.traintest
:   Class

ResultCheckerTest(TableRow) - Constructor for class test.ml2db.mulan.learn.traintest.ResultCheckerTest


RESULTS\_DIRECTORY - Static variable in class main.ml2db.ProjectParameters


RESULTS\_DIRECTORY - Static variable in class test.ml2db.al.ActiveLearningTest


RESULTS\_DIRECTORY\_ACTIVE\_LEARNING - Static variable in class main.ml2db.ProjectParameters


RESULTS\_DIRECTORY\_ACTIVE\_LEARNING - Static variable in class test.ml2db.dataharness.TestProjectParameters


RESULTS\_FOLDER - Static variable in class test.ml2db.dataharness.TestProjectParameters


ResultsFormatterTest - Class in test.ml2db.mulan.learn.database
:   Class

ResultsFormatterTest() - Constructor for class test.ml2db.mulan.learn.database.ResultsFormatterTest


run() - Method in class main.ml2db.al.ActiveLearning


run(NeighbourhoodNetwork) - Static method in class main.ml2db.al.utility.neighbours.NeighbourhoodExperimentDetailsSerialised


run() - Static method in class main.ml2db.graphviz.GraphVizPrinter


run(int, int, DbManagers) - Static method in class main.ml2db.mulan.attributesfiltered.AttFilter
:   Filter the old test arff by the train arff given and regenerate the old
    train set using all train+test classes.

run(int[], String, int, Vector<MulanLearner>, boolean, boolean, boolean, int) - Static method in class main.ml2db.mulan.learn.crosseval.CrossExperimenter
:   Run a cross-evaluation of several ARFF files (using one or several
    algorithms, as needed)

RUN - Static variable in class main.ml2db.mulan.learn.crosseval.EvaluationResultsTable
:   the evaluation run number (eg: between 0 and 9 for a 10 fold cross
    evaluation)

run() - Method in class main.ml2db.mulan.learn.Experimenter


run(int, MulanLearner, DbManagers, String) - Static method in class main.ml2db.mulan.learn.MulanSerializer


run() - Static method in class main.ml2db.mulan.learn.traintest.TrainTestExperimenter


run(String, DbManagers, boolean) - Static method in class main.ml2db.mulan.mulanarff.MulanArff
:   Generates the ARFF (and XML) files and returns the identifier of the arff
    record in the database

run(String[], DbManagers, boolean) - Static method in class main.ml2db.mulan.mulanarff.MulanArff
:   Generates a number of ARFF files (the database the records are written is
    the same for all ARFF files)

run(String, DbManagers, boolean) - Static method in class main.ml2db.weka.Arff


runActiveLearningStep(int) - Method in class main.ml2db.al.ActiveLearning
:   Calculate utility metric for each instance, pick best (query instance),
    add query instance to labelled set and delete from unlabelled set.

runFromSerialised() - Static method in class main.ml2db.graphviz.GraphVizPrinter


runWithSerializedTrainedModel(DbManagers, int, int, MulanLearner, String, String, boolean, boolean) - Static method in class main.ml2db.mulan.learn.traintest.TrainTestExperimenterSerialized

A B C D E F G H I J K L M N O P Q R S T U V W X

- Overview
- Package
- Class
- Use
- Tree
- Deprecated
- Index
- Help

- Prev Letter
- Next Letter

- Frames
- No Frames

- All Classes
